# Coupling Effect of Molecular Chain Displacement and Carrier Trap Characteristics on DC Breakdown of HDPE/LDPE Blend Insulation

**DOI:** 10.3390/polym12030589

**Published:** 2020-03-05

**Authors:** Zhonglei Li, Mingsheng Fan, Zhuoyan Zhong, Boxue Du

**Affiliations:** Key Laboratory of Smart Grid of Education Ministry, School of Electrical and Information Engineering, Tianjin University, Tianjin 300072, China; 18428330798@163.com (M.F.); zhuoyan@tju.edu.cn (Z.Z.); duboxue@tju.edu.cn (B.D.)

**Keywords:** polyethylene, dielectric relaxation, DC breakdown, molecular chain displacement, trap characteristics

## Abstract

This work focuses on the coupling effect of molecular chain displacement and trap characteristics on direct current (DC) breakdown properties of high density/low density polyethylene (HDPE/LDPE) blend insulation. Frequency domain spectroscopy (FDS) and isothermal discharge current (IDC) are used to characterize the dielectric relaxation and trap characteristics of HDPE/LDPE blends. A DC breakdown model is proposed to reveal the mechanisms of the molecular chain displacement and carrier trap on the DC breakdown strength. The dielectric relaxation α and δ present segmental motions and thermal ion polarization behaviours of HDPE/LDPE blends, respectively. α dielectric relaxation strength (Δ*ε*_α_) increases as the amount of HDPE increases from 0 to 5 wt%, and then declines with a further increase of HDPE content to 20 wt%. According to the velocity equation, the increase of Δεα will increase the molecular chain displacement, resulting in a larger free volume, which will provide electrons with larger free path λ to form hot electrons. A positive correlation exists between the activation energy of the dielectric relaxation process δ and trap density, and the increase of δ dielectric relaxation strength (Δ*ε*_δ_) will adversely affect the breakdown strength of the specimen. HDPE/LDPE blends with 15 wt% HDPE content have lower Δ*ε*_α_ and lowest Δ*ε*_δ_, which decreases the mean free path λ of molecular chain and thermal ion polarization. At the same time, it has the highest deep trap density, thus increasing the probability of hot electrons being captured and improving the DC breakdown strength. It is concluded the breakdown of the dielectric is synergistically affected by the molecular chain displacement and carrier trap.

## 1. Introduction

Cross-linked polyethylene (XLPE) insulation cables are widely used and developed in high-voltage power transmission projects due to their advantages such as light weight, high operating temperature, and high transmission power [1,2]. However, with the large-scale use of XLPE as a cable insulation material, many problems and technical difficulties in the production, operation, and recycling of XLPE cables have gradually emerged. In addition, the by-products introduced in the cross-linking process need to be degassed for a long time, which reduces the production efficiency [3,4]. Therefore, the development of thermoplastic, non-crosslinking cable materials is the future trend of power cable development [5].

As an environmentally friendly material, polyethylene is widely used as the insulation of power cables, which are considered to have excellent prospects [6,7]. Chen et al. found that PE-based materials may actually show excellent performance in alternating current (AC) applications, but show certain performance limits under DC conditions [8]. Adding certain thermoplastic insulation materials to LDPE matrix through physical blending can effectively improve the insulation and mechanical properties. Lin et al. found that blending HDPE in LDPE is helpful for nucleation of matrix crystals, which reduces the average size of spherulites. Smaller spherulites lead to an increase in the interface between the crystalline phase and the amorphous phase, which improves the charge transfer characteristics and reduces the space charge [9]. Andersson et al. found that blending a small amount of HDPE in LDPE will reduce the DC conductivity and improve the mechanical properties under high temperature fields [10].

Deep traps are known to play a crucial role in the breakdown performance of polymer [11,12]. Deep traps in polymers mainly originate from crystal interfaces, which can capture a large number of free carriers [13]. Wang et al. found that the enhanced height of barrier, the decrease of mobility of carriers and the formation of homocharges caused by deep traps improved breakdown performance of polymers [14]. Jiang et al. found that the deep traps at the interface had a significant impact on the space charge trapping process and enhance DC breakdown performance of polymers [15]. Moreover, many references have demonstrated that the insulation properties of polymers are affected by the molecular chain displacement [16,17,18]. Min et al. proposed a model combining the charge transport characteristics and molecular motion to characterize the breakdown mechanism of LDPE, and the breakdown phenomenon occurs when the displacement of the molecular chain reaches a certain threshold [19]. Xie et al. found that an increase in temperature will aggravate the movement of molecular chains in the polymer and increase the energy of the carriers, which will eventually lead to a decrease in the breakdown performance of the dielectric [20].

In this study, the DC breakdown, dielectric relaxation, and carrier trap characteristics of HDPE/LDPE blend insulation are investigated. A relationship between the dielectric relaxation and molecular chain displacement is investigated, and a DC breakdown model is proposed to reveal the coupling effect of the molecular chain displacement and carrier trap on the DC breakdown strength.

## 2. Experimental Arrangement

### 2.1. Process of Samples Preparation

HDPE / LDPE blends samples were prepared by melt blending, using LD100BW low density polyethylene from Beijing Yanshan Petrochemical Company (Beijing, China) and L501 high density polyethylene from Beijing Yanshan Petrochemical Company (Beijing, China). The specimen preparation method used in this article is shown below. The melt blending was conducted in a twin-screw continuous mixer rotation (YZK-4, Yizong Instrument Co., Ltd., Dongguan, China) and the test specimens were prepared by hot press forming machine (DY-30, Jingsheng Scientific Instrument Co., Ltd., Shanghai, China). First, HDPE and LDPE should be dried at 50 °C for 6 h before the experiment. Second, HDPE and LDPE should be blended into a 175 °C blender for 20 min in a certain proportion, and the ratio of HDPE / LDPE is 0 wt%, 5 wt%, 10 wt%, 15 wt%, 20 wt%, respectively. Third, the above blended materials are put into a stainless-steel mold and pressed at a pressure of 10 MPa for 10 min under a 175 °C environment. Finally, samples with thicknesses of 85 µm and 300 µm were obtained, which were used to test the DC breakdown strength and dielectric properties, respectively. The hot-press forming process can effectively control the thickness difference of the samples to not exceed 5 µm, and the temperature and time during hot pressing are strictly controlled to ensure that the morphology are basically the same. The same PET film is used as the gasket on the sample film to ensure the same surface morphology.

### 2.2. Experimental Methods

The real and imaginary parts of the permittivities of the samples at 40, 60 and 80 °C are measured using the frequency domain spectroscopy (FDS) method (Concept40, Novocontrol Technologies, Frankfurt, Germany). Aluminum foil is affixed to both sides of the sample, and the sample was is placed between the electrodes, and the measurement voltage is 1 kV. The test frequency varied from 10^−2^ to 10^4^ Hz.

**Figure 1:**
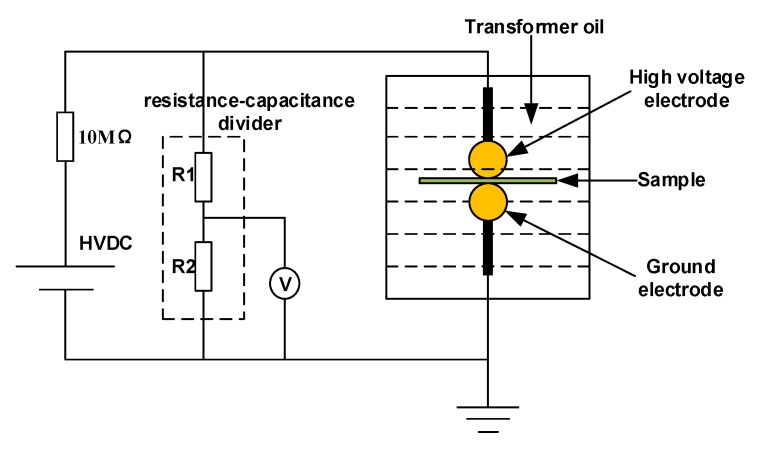
The schematic diagram of DC breakdown test.

The DC breakdown field strength of samples in this article is tested by the ball electrode method. The schematic diagram of DC breakdown test is shown in Figure 1. In order to prevent flashover occurs during the breakdown test of the electrode and the sample edge, the electrode and samples in this device are immersed in transformer oil. The voltage is linearly increased at a step-up rate of 0.5 kV/s, until a sample of 85 µm thickness breaks down. The voltage at this time is recorded as the breakdown voltage, and the breakdown field strength is obtained from the sample thickness at this voltage. We did breakdown tests 15 times at different locations on the same specimen.

**Figure 2:**
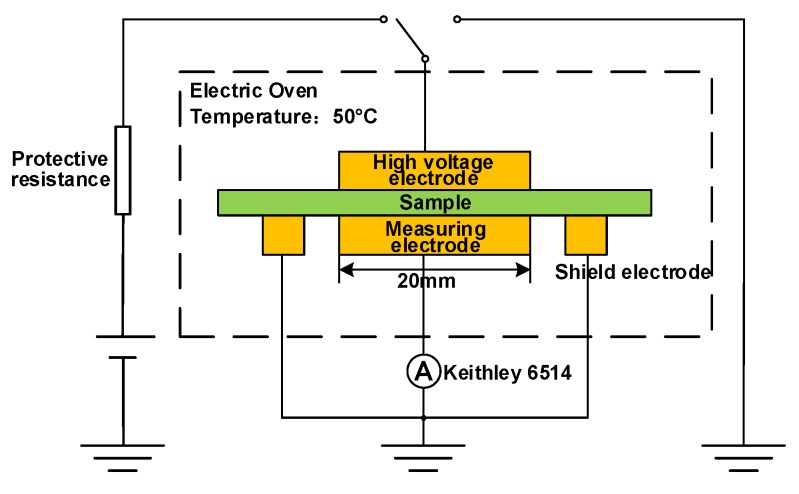
The schematic diagram of IDC test.

In this paper, the trap level distribution of the five HDPE/LDPE blends is calculated by isothermal discharge current (IDC) method [21], as is shown in Figure 2. Al electrodes were deposited on both sides of polymer film specimens to ensure good contact between the specimen and the measuring electrode. The sample is placed between two stainless steel electrodes with a diameter of 20 mm, as shown in Figure 1. The five samples are polarized for 45 min under two 20 mm diameter electrodes, and the applied voltage is 30 kV/mm and the temperature is 50 °C. The samples are then shorted on both sides, and the depolarization current of samples are collected for 30 min using an ammeter. The IDC experiments of the five samples in this study were measured three times.

### 2.3. Trap Distribution Characterization

IDC is a method proposed by Simmons in 1973 to study the density distribution of trap energy level in materials, and the density of traps (*N_t_*(*E*)) and the traps energy (*E_t_*) can be derived from the following equations: [21]:

(1) $$N_{t}(E)=\frac{2dIt}{ql^{2}kT}$$

(2) $$E_{t}=kT\ln(vt)$$

where *l* is the depth of charge injection and *d* is the thickness of samples in this paper. *q* represents the standard charge amount and *k* is the Boltzmann’s constant, *k* = 1.38 × 10^−23^ J/K. *T* is the absolute temperature, where T is 323.15 K. *v* is the escape frequency of trapped charges, and its value is ~10^12^ s^−1^. In order to improve the fitting of discharge current data for short times, two exponential decay function has been used for a better approximation of the experimental results.

## 3. Results and Analysis

### 3.1. Dielectric Constant and Dielectric Loss

**Figure 3:**
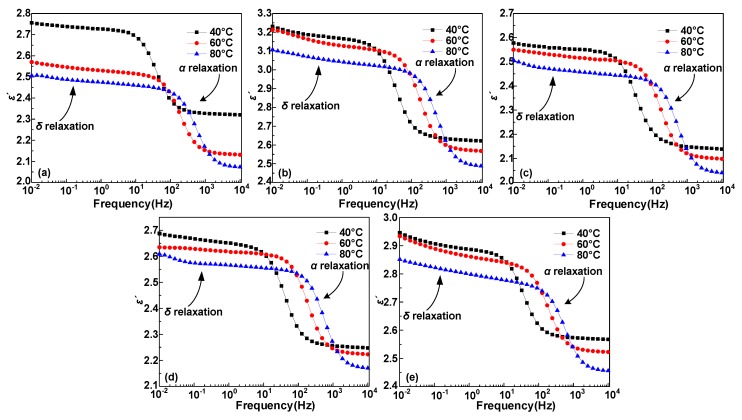
The relationship between real part of permittivity *ε*′ and frequency at 40, 60 and 80 °C for HDPE/LDPE blends with different HDPE contents (**a**) 0 wt%, (**b**) 5 wt%, (**c**) 10 wt%, (**d**) 15 wt%, and (**e**) 20 wt%.

**Figure 4:**
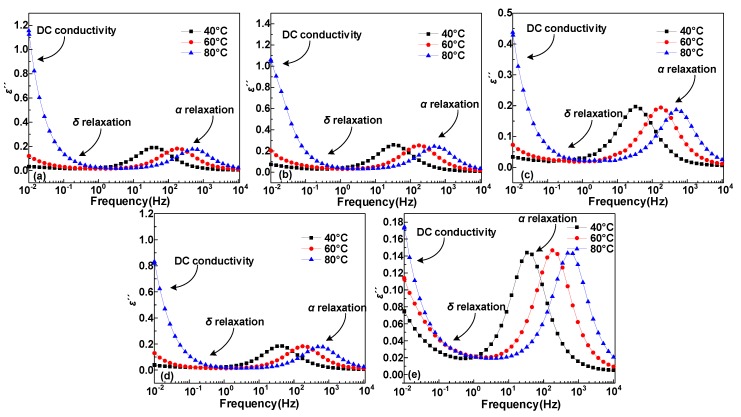
The relationship imaginary part of permittivity *ε*″ and frequency at 40, 60 and 80 °C for HDPE/LDPE blends with different HDPE contents (**a**) 0 wt%, (**b**) 5 wt%, (**c**) 10 wt%, (**d**) 15 wt%, and (**e**) 20 wt%.

Dielectric constant (*ε*′) and dielectric loss (*ε″*) test results of five samples at 40, 60, and 80 °C are shown in Figure 3 and Figure 4, where the test frequency is from 10^−2^ to 10^4^ Hz, where α and δ relaxation processes occur at high and low frequencies, respectively. Frist, both the *ε*′ and *ε″* results show a remarkable relaxation peak above 10 Hz, which is *α* relaxation process, and the *α* relaxation is closely relevant to the displacement of molecular segments in the polymer [22,23]. Then, we can find that both *ε*′ and *ε″* spectrum have an upward process when the frequency continues to decline. This process is the *δ* relaxation process, which is related to the localized states hopping behavior of the carrier in the polymer [23]. In addition, for the *ε*′ and *ε″* spectrum of the same sample, the spectral peaks will move towards a higher frequency and the *ε″* spectrum shows a distinct upswing process during the temperature rise process. Hence, this process mainly causes dielectric loss [23,24].

**Figure 5:**
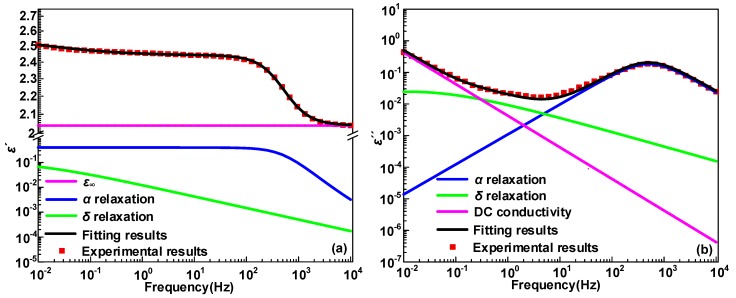
(**a**) The fitting and actual results for the *ε*′ and (**b**) *ε″* of HDPE/LDPE blends with 15 wt% HDPE content at 80 °C. The curves represent the fitting results and symbols are the actual experimental results.

According to the Havriliak–Negami (H-N) function equation, the *ε*′, *ε″* of *α*, *δ* and DC conductivity processes are well fitted. The H-N function equation is expressed as

(3) $$\varepsilon^{*}=\varepsilon_{\infty }+\frac{\Delta \varepsilon_{\alpha }}{{\left(1+{\left(i\omega \tau_{\alpha }\right)}^{\beta_{\alpha }}\right)}^{\gamma_{\alpha }}}+\frac{\Delta \varepsilon_{\delta }}{{\left(1+{\left(i\omega \tau_{\delta }\right)}^{\beta_{\delta }}\right)}^{\gamma_{\delta }}}+\frac{\sigma }{i\varepsilon_{0}\omega }$$

where *ε*_∞_ is the optical dielectric constant, and the relaxation time constants of ε_∞_ are usually within the scope of 10^−^^16^~10^−^^15^ s and 10^−^^14^~10^−^^13^ s [24]. *ω* represents the oscillation frequency of electrical field. Δ*ε*_α_ and *τ_α_* represents the dielectric relaxation strength and the relaxation time constant, respectively. *β_α_* and *γ_α_* are shape coefficients, while Δ*ε*_δ_, *τ_δ_*, *β_δ_*, and *γ_δ_* are similar to the parameters for dielectric relaxation *α*. *ε*_0_ is the dielectric constant under vacuum and *σ* is relevant to DC conductivity. The experimental results and fitting results of *ε*′ and *ε″* spectrum of HDPE/LDPE blends with 15 wt% HDPE content at 80 °C is illustrated in Figure 5, which implies the curves calculated by the H-N equation can fit the experimental results well.

### 3.2. Trap Level Characteristics

**Figure 6:**
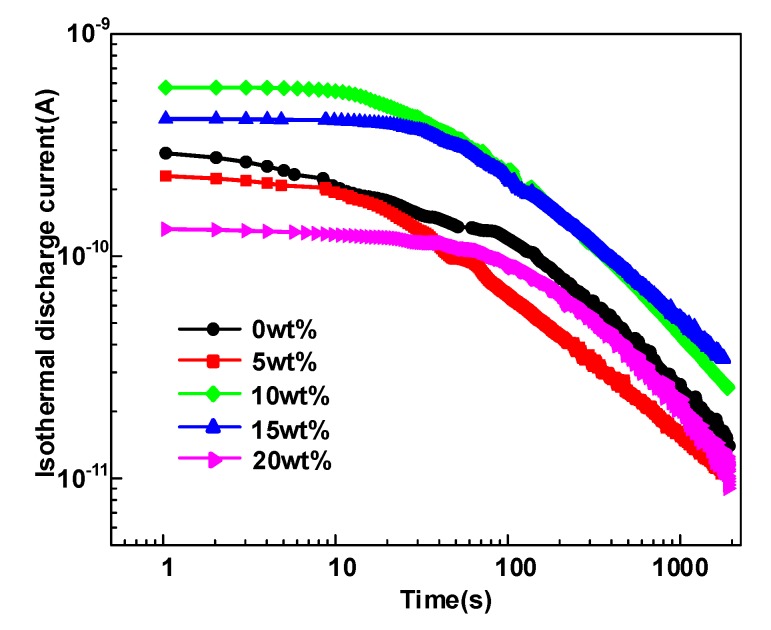
Isothermal discharge current of HDPE/LDPE blends obtained by IDC method.

**Figure 7:**
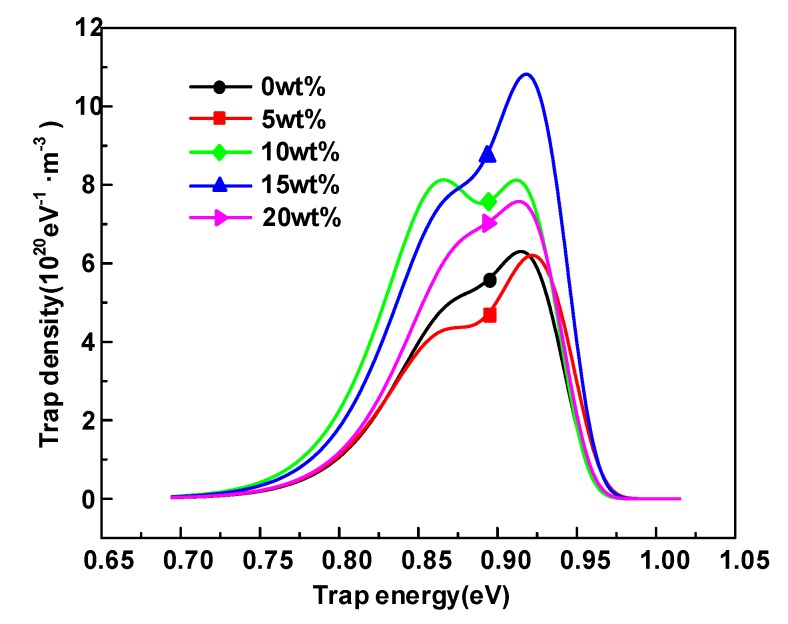
Trap level distribution of HDPE/LDPE blends obtained by IDC method.

The isothermal discharge current and the trap levels distributions of HDPE/LDPE blends are obtained and illustrated in Figure 6 and Figure 7. It is obviously observed that the deep trap level of HDPE/LDPE blends is in the range of 0.92~0.93 eV. Deep traps density keeps an upward tendency with the content of HDPE from 0 to 15 wt%, and then decreases when the content of HDPE increases to 20 wt%. The density of shallow traps roughly has the similar trends with the deep traps. The density of deep trap is closely relevant to the carrier migration in polymers, so this paper will focus on the effect of deep traps on the dielectric breakdown strength.

### 3.3. DC Breakdown Characteristics

**Figure 8:**
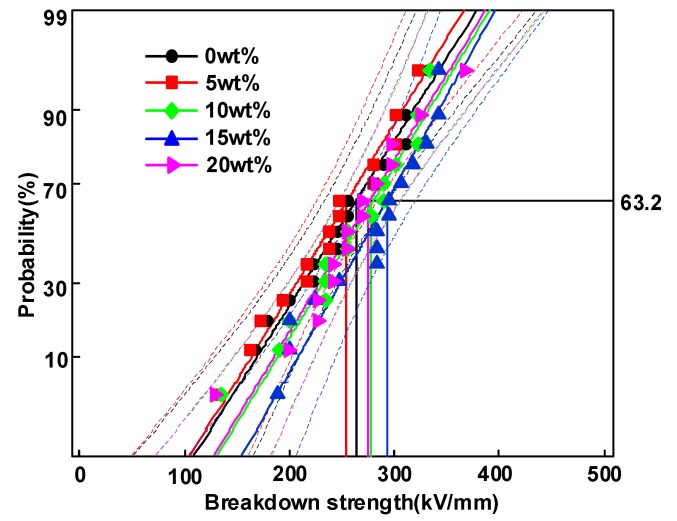
Breakdown strength results of HDPE/LDPE blends at 40 °C.

Figure 8 demonstrates the electrical breakdown test results of HDPE/LDPE blends under 40 °C. *E*_b_ is the breakdown strength at a breakdown possibility of 63.2%. *E*_b_ is 263.2, 254.7, 276.6, 293.8 and 276.1 kV/mm for HDPE/LDPE blends with HDPE content from 0 to 20 wt%, respectively. The addition of HDPE first reduce and then induce the DC breakdown performance of HDPE/LDPE blends, *E*_b_ reaches the highest value at 15 wt% then declines when the HDPE content increases to 20 wt%. The distribution parameter of the Weibull plots is 10.1, 10.1, 10.9, 11.8 and 10.4 for HDPE/LDPE blends with HDPE content from 0 to 20 wt%, respectively.

## 4. Discussion

### 4.1. Dipole Motion Relaxation

**Figure 9:**
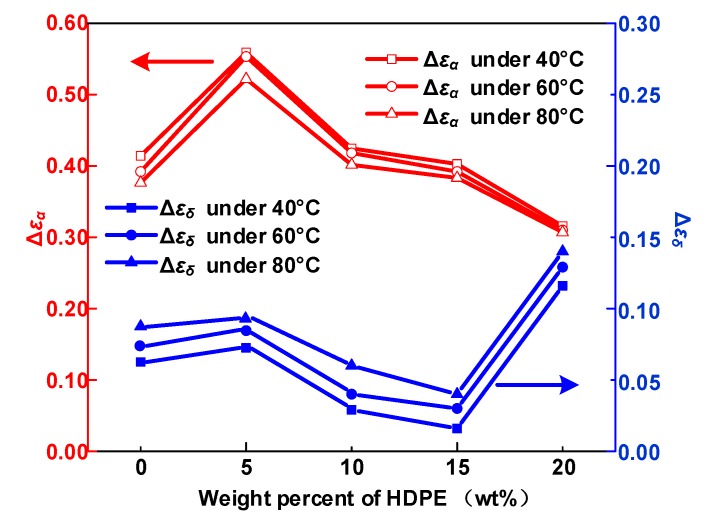
*α* Relaxation strength (Δ*ε*_α_) and *δ* relaxation strength (Δ*ε*_δ_) of HDPE/LDPE blends at 40, 60 and 80 °C.

The calculated results for *τ*_α_, *τ*_δ_, Δ*ε*_α_, Δ*ε*_δ_ and *σ*_DC_ are obtained from Equation (3). Figure 9 demonstrates the *α* relaxation strength (Δ*ε*_α_) and *δ* relaxation strength (Δ*ε*_δ_) of five samples at 40, 60, and 80 °C.

The *α* relaxation is relevant to the movement of molecular segments in amorphous phase [25]. *α* relaxation is caused by the orientation of the dipoles in the molecular chain under the action of microscopic Brownian motion [26]. The increase in temperature allows the dipoles to obtain enough energy to be aligned under the action of the electric field, thereby increasing the dielectric constant. Nevertheless, further increases in temperature will exacerbate the thermal Brownian motion, leading to a random movement of a large number of dipoles, preventing them from being aligned in the direction of the electric field. Hence, Δ*ε*_α_ declines with temperature as shown in Figure 8. Δ*ε*_δ_ keeps an upward tendency with the temperature, because carriers get more energy and the motion of carriers is easier in HDPE/LDPE blends.

The *α* relaxation process is closely related with the dipoles in polymer, which is coincident with the crystallinity of samples. In addition, Δ*ε*_α_ also depend on the number of dipoles in the amorphous area [26]. Polymer contains more dipoles as well as lower crystallinity have higher Δ*ε*_α_. Δ*ε*_α_ increases as the amount of HDPE increases from 0 to 5 wt%, and then decreases when the HDPE content increases to 20 wt%. Thus, it is inferred that the sample with 5 wt% content HDPE has the highest molecular chain alignment disorder, looseness, and flexibility, so that molecular chains can easily be rearranged under the action of an electric field to form a void segment.

### 4.2. Thermal Ion Polarization Behaviours

The dielectric relaxation process *δ* involves hopping polarization [23]. Localized charges (ions or vacancies) hop among the localized states, leaving opposite polarity charges at different positions to form dipoles [22].

**Figure 10:**
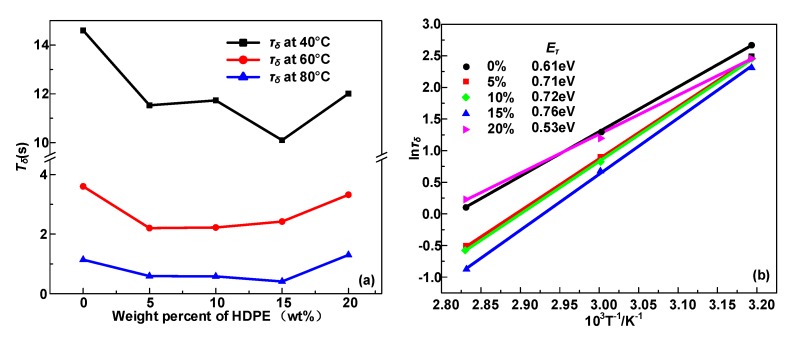
(**a**) *δ* relaxation time constant (τ_δ_) at 40, 60 and 80 °C of HDPE/LDPE blends. (**b**) The relaxation time constant (ln(τ_δ_)) as a function of the reciprocal of the absolute temperature for HDPE/LDPE blends.

The relationship between *τ*_δ_ and absolute temperature is fitted by the following equation, and the required energy for hopping polarization is calculated [27].

(4) $$\tau_{\delta }\left(T\right)=\tau_{0}\exp\left(-\frac{E_{\tau }}{kT}\right)$$

where *τ*_0_ is a constant, *E*_τ_ represents the activation energy of hopping polarization, and *k* represents the Boltzmann’s constant. The *δ* relaxation time constant and Arrhenius plot is illustrated in Figure 10. τ_δ_ is in the range of 10^−1^~10^2^ s, so this process refers to hopping polarization [22]. The fitting parameters *E*_τ_ of HDPE/LDPE blends with HDPE content from 0 to 20 wt% are 0.61, 0.71, 0.72, 0.76, and 0.53 eV, respectively.

The trap level is potential barriers that carriers need to overcome to jump between localized states. With the increase of trap density in HDPE/LDPE blends, the activation energy in δ relaxation process also increase, since the increase of trap density will also cause more homopolar charges to be confined on the surface of the dielectric, and the electric field generated by these homopolar charges (in the opposite direction to the externally applied electric field) will reduce the carrier injection equivalent electric field [28], eventually increasing the activation energy in the δ relaxation process. The carrier required detrapping energy level (*Φ*_eff_) in polymer is positively correlated to the trap level of the localized states (*Φ*_t_) [28].

(5) $$\phi_{\mathrm{eff}}=\phi_{t}-\frac{1}{2}eE_{ext}\lambda$$

where *E*_ext_ represents the external field, and *λ* represents the mean free path of charges. *Φ*_eff_ represents the potential barrier for trapped electrons to overcome for hopping from localized states. Equation (5) implies that a deeper trap level has a higher potential barrier in hopping polarization behavior. Moreover, increasing the density of deep traps increases the possibility of trapping during carrier migration, enhances trap trapping effects and reduces the mean free path of carriers [29]. Thus, more energy will be required for hopping polarization with the growth of the deep traps density.

**Figure 11:**
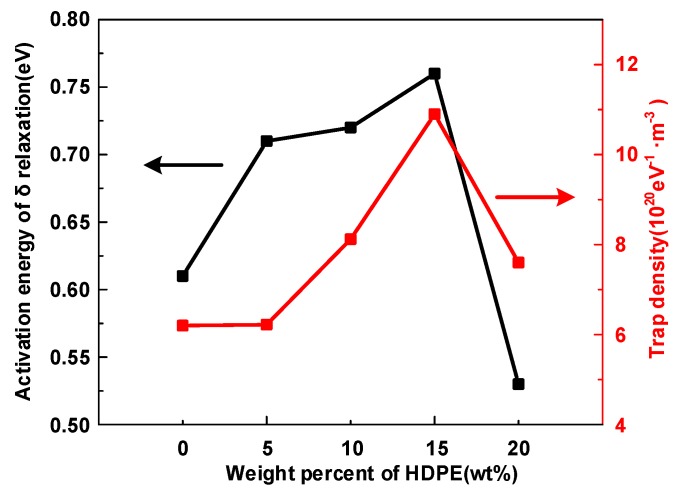
The activation energy of the δ relaxation and trap density of HDPE/LDPE blends.

In addition, Δ*ε*_δ_ has an upward tendency with the content of HDPE from 0 to 5 wt%, then declines when HDPE content increases to 15 wt%, but when the HDPE content reaches 20 wt%, Δ*ε_δ_* attains the highest value. Figure 11 is the activation energy of the *δ* relaxation and trap density of HDPE/LDPE blends, and both the activation energy and trap density show the opposite trend from Δ*ε_δ_* in Figure 9 with HDPE content increase from 0 to 20 wt%. It is remarkable that when the added content of HDPE reaches 15 wt%, the sample has the highest breakdown strength, at the same time it has the lowest Δ*ε_δ_* and the highest deep trap density and activation energy of the *δ* relaxation.

### 4.3. Electrical Breakdown Model of Coupling Molecular Chain Displacement and Carrier Trap Characteristics

**Figure 12:**
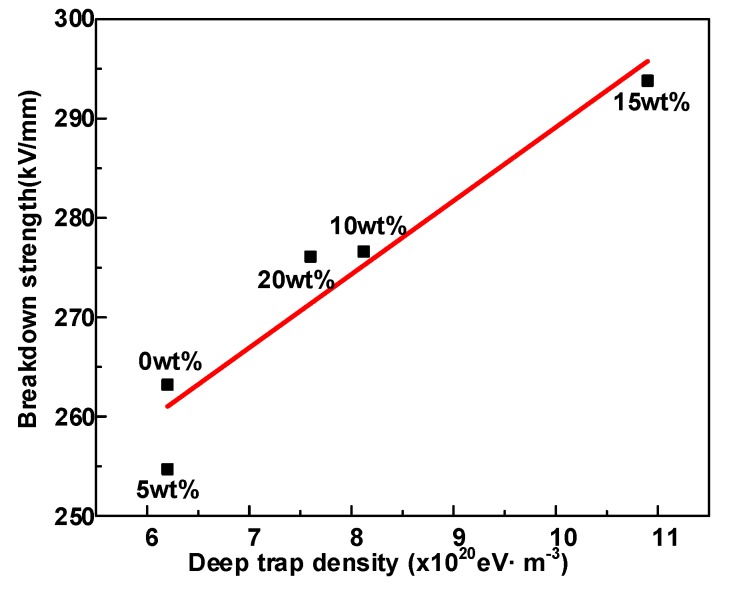
DC Breakdown field at 40 °C as a function of the deep trap density.

Previous works [29] showed that the increase in the energy level and density of deep traps will have an excellent effect on the breakdown performance of electrical insulating dielectrics. Hence, the effect of deep traps on the breakdown strength of samples will be studied in conjunction with molecular chain displacement in this section. Figure 12 depicts the relationship between breakdown strength and deep trap density. The plot indicates that the HDPE/LDPE blends with high deep trap density exhibit higher breakdown strengths.

It is assumed that free volumes exist in HDPE/LDPE blends, and electrons can accumulate energy under the electric field and not dissipate. The breakdown mechanism can be demonstrated as follows [30].

(6) $$E_{critical}=\frac{E_{t}}{e\lambda_{e}}$$

As a consequence, the mean free path of electrons (*λ_e_*) and deep potential barriers (*E_t_*) cooperate to affect the dielectric critical breakdown field strength (*E_critical_*), and the deep trap level represents *E_t_* [29,31], which is in the range of 0.92~0.93 eV. The increase of deep traps level can enhance *E_critical_*, since higher barriers limit the transport and migration of free carriers. All samples in this paper have similar deep trap energy levels as shown in Figure 6, so the electron mean free path is the main factor affecting the breakdown field strength of the sample.

The density of deep traps has a remarkable influence on the free carrier migration [30], and the increase of deep trap density also enhances the hot electrons trapping possibility and decline the *λ_e_*, and the trapping possibility of charges can be expressed by [31,32]

(7) $$P_{tr}=eN_{t}\mu_{0}/\varepsilon_{0}\varepsilon_{r}$$

where *P_tr_* is the trapping possibility of charges in s^−1^, *μ*_0_ is carrier mobility, and *ε_r_* is the relative permittivity.

Nevertheless, according to Equation (6), the longest electron mean free path *λ_e_* is about 3.1~3.5 nm when the breakdown occurs, which is obviously not equal to the mean free path inherent in polyethylene (<1 nm). Moreover, HDPE/LDPE blends with 0 wt% and 5 wt% HDPE mass fraction samples have almost similar deep trap levels and densities, but the sample with HDPE content of 5 wt% has a lower breakdown strength. Therefore, the investigation of the breakdown mechanism needs to consider the free path of the charge generated by the displacement of the molecular segment.

**Figure 13:**
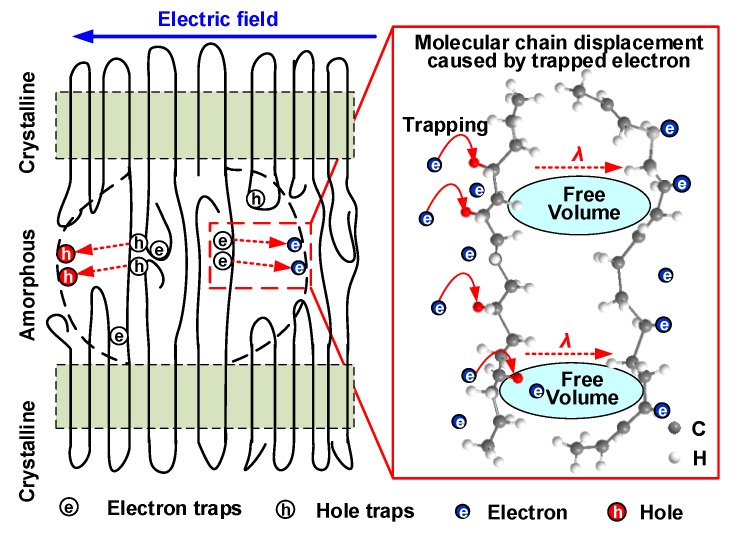
Schematic model for illustrating the molecular chain displacement caused by trapped carriers under the DC electric field.

Hence, a molecular chain displacement model was established, as shown in Figure 13. It has been widely confirmed that the molecular chains are arranged in parallel and orderly as crystalline regions, while those with loose and irregular aggregation are amorphous regions. The chemical defects on molecular chains can act as deep traps [33,34], electrons can penetrate into the loose amorphous region [35], and these electrons will have a certain possibility of being captured by the deep traps. Moreover, it is difficult for an electron captured by a deep trap to get enough energy to jump out of the deep trap and become a free carrier [36], so the charge on the molecular chain will be subjected to a Coulomb force for a sufficient time on account of the long residence time of deep trapped electrons [37,38]. The trap residence time (*τ*_trap_) of traps of different energy levels might be several orders of magnitude, which can be expressed as [39]

(8) $$\tau_{\mathrm{trap}}=v^{-1}\exp(E_{t}/k_{B}T)$$

Hence, the trap residence time constant is approximately 10^−13^ s when trap level is 0.1 eV, and trap residence time is about 500 s for 1.0 eV traps [40,41].

Hence, deep traps play a major role in the molecular chain displacement process. This phenomenon of molecular chain displacement process can be further investigated by the following velocity equation [42].

(9) $$\frac{d\lambda }{dt}=\mu_{0}E_{appl}-\frac{\lambda }{\tau_{\alpha }}$$

where *λ* is the molecular chain displacement in m, *E*_appl_ is applied field in V/m.

Therefore,

(10) $$\lambda =\mu_{0}\tau_{\alpha }E_{appl}+Cexp(-\frac{t}{\tau_{\alpha }})$$

where *C* stands for a constant. Here the carrier mobility *μ*_0_ is assumed to be in the range from 1 × 10^−15^ to 5 × 10^−15^ m^2^V^−1^s^−1^ [43]. The values of *τα* at 40 °C calculated by Equation (3) are shown in Table 1, and the part of C·exp(−t/τα) approach zero with the increase of polarization time. Hence, after a sufficient period of relaxation under an applied electric field close to DC breakdown field strength, the value of molecular chain displacement is in the range of 1.13 to 6.67 nm, which corresponds to the results calculated by Equation (6).

In this case, it can be considered that charged molecular chain is formed, which will be displaced by the electric field, the increase of Δ*ε*_α_ will increase the displacement of the molecular chain, thereby forming many larger free volumes. The larger free volume will make the electrons accelerate to get more energy under the action of the electric field, which may then become hot electrons. In the Kao’s model [44], the chain scission is caused by hot electrons that continuously impact the molecular chain, and this process continues and implies that a low density region is formed. Thus, the subsequent hot electron impact on the molecular chain may cause the molecular chain to rupture and reduce the breakdown strength of the sample.

**Figure 14:**
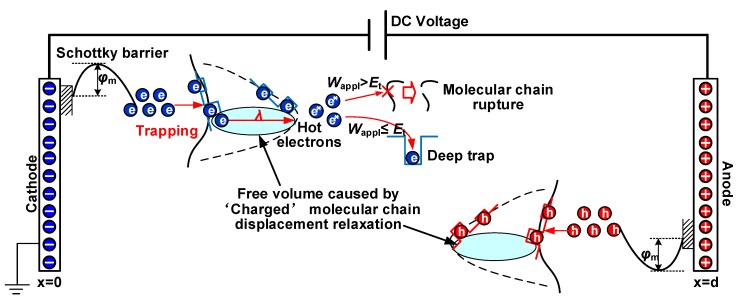
The breakdown model of coupling carrier trap characteristics and molecular chain displacement.

Hence, a breakdown model combining the molecular chain displacement and carrier trap was established as shown in Figure 14. Electrons injected from the cathode through Schottky barrier can accelerate and accumulate energy in a free volume. The increase in free volume caused by α relaxation enhances the free path of electrons and thus electrons can gain more energy to form hot electrons. The accumulated energy of charges in an electric field is synergistically affected by the molecular chain displacement and electrical field strength, which can be illustrated as [31,45].

(11) $$W_{appl}=eE_{appl}\lambda$$

If the hot electrons with an energy of *W*_appl_ are smaller than the deep potential barrier *E*_t_, these electrons will be captured by deep traps. On the other hand, these hot electrons with energy as *W*_appl_ are larger than the potential barrier *E*_t_, they can directly cross over deep traps and constantly hit the molecular chain. Moreover, the growth of deep trap density *N*_t_ will enhance the possibility of hot electrons being captured [46], preventing a large number of hot electrons from directly hitting the molecular chain and causing the molecular chain to rupture, thereby improving the DC breakdown field strength of the HDPE/LDPE blends.

## 5. Conclusions

HDPE/LDPE blends are prepared to study the coupling effect of molecular chain displacement and carrier trap characteristics on their DC breakdown strength, and the following conclusions have been drawn.

1. The *α* and *δ* relaxation in HDPE/LDPE blends turn out to contain the molecular segments displacement and carrier’s hopping, respectively. Δ*ε*_α_ declines with the increase of temperature, because of random thermal motion of dipoles caused by Brownian motion. Δ*ε*_δ_ increases with the temperature, since higher temperatures provide more energy for the carriers to hop polarization. The activation energy of the dielectric relaxation process *δ* is positive related to the trap density obtained by IDC test.
2. Δ*ε*_α_ increases as the amount of HDPE increases from 0 to 5 wt%, then declines with a further increase of HDPE content to 20 wt%. The increase of Δ*ε*_α_ will increase the displacement of the molecular chain inside the sample, resulting in a larger free volume, which will provide electrons with larger mean free path to obtain energy, and eventually continue to hit the molecular chain to cause breakdown.
3. Δ*ε*_α_ and deep trap density *N*_t_ synergistically influence the average free path of the carrier and ultimately affect the breakdown performance of the dielectric. Moreover, the growth of deep traps density decreases the mean free path of electrons and prevents hot electrons from hitting the molecular chain directly, leading to an improvement in the DC breakdown field.

## Figures and Tables

**Table 1:** The value of *τ_α_* at 40 °C.

Weight Percent of HDPE (wt%)	τ_α_ at 40 °C
0	4.32 × 10^−3^
5	5.05 × 10^−3^
10	4.81 × 10^−3^
15	4.42 × 10^−3^
20	4.35 × 10^−3^
